# Estimating number of cases and spread of coronavirus disease (COVID-19) using critical care admissions, United Kingdom, February to March 2020

**DOI:** 10.2807/1560-7917.ES.2020.25.18.2000632

**Published:** 2020-05-07

**Authors:** Mark Jit, Thibaut Jombart, Emily S Nightingale, Akira Endo, Sam Abbott, LSHTM Centre for Mathematical Modelling of Infectious Diseases COVID-19 Working Group, W John Edmunds

**Affiliations:** 1 Centre for Mathematical Modelling of Infectious Diseases, Department of Infectious Disease Epidemiology, London School of Hygiene and Tropical Medicine, London, United Kingdom; 2 School of Public Health, Li Ka Shing Faculty of Medicine, University of Hong Kong, Hong Kong SAR, China; 3 United Kingdom Public Health Rapid Support Team, London, United Kingdom; 4 MRC Centre for Global Infectious Disease Analysis, Department of Infectious Disease Epidemiology, School of Public Health, Imperial College London, United Kingdom; 5 The members of the LSHTM Centre for Mathematical Modelling of Infectious Diseases COVID-19 Working Group are listed at the end of this article

**Keywords:** coronavirus disease 2019, SARS-CoV-2, intensive care unit, surveillance, reproduction number, mathematical model

## Abstract

An exponential growth model was fitted to critical care admissions from two surveillance databases to determine likely coronavirus disease (COVID-19) case numbers, critical care admissions and epidemic growth in the United Kingdom before the national lockdown. We estimate, on 23 March, a median of 114,000 (95% credible interval (CrI): 78,000–173,000) new cases and 258 (95% CrI: 220–319) new critical care reports, with 527,000 (95% CrI: 362,000–797,000) cumulative cases since 16 February.

The first reported cases of coronavirus disease (COVID-19) in the United Kingdom (UK) were identified in late January 2020 [1]. By 23 March, when a national lockdown prohibiting non-essential movement was announced, reported cases had increased to over 6,000. However, reported cases are probably a small fraction of total infections because most COVID-19 cases are mild or even asymptomatic [2]. Also, the proportion of infections that are reported changes by setting and over time, depending on the intensity of population-based surveillance, testing and contact tracing. However, COVID-19 patients admitted to critical care (high-dependency or intensive care units, hereafter CC) offer a more stable indicator, since CC patients suspected of having COVID-19 have always been tested in the UK. We used the two COVID-19 CC surveillance databases available at the start of the epidemic, combined with data on reporting delays and disease severity to determine the likely number of and increase in infected people during the first phase of the COVID-19 epidemic in the UK. This work informed estimates produced by the Scientific Pandemic Influenza Group on Modelling (SPI-M) to advise the UK’s Department for Health and Social Care on its COVID-19 response.

## Data sources

Two data sources were used for CC case numbers:

(i) Public Health England (PHE) compiled the First Few Hundred (FF100) database containing virologically confirmed COVID-19 cases in the UK during the first phase of the epidemic [3]. We extracted ‘sporadic’ cases which were those identified through CC surveillance rather than contact tracing or targeted testing of travellers from high-risk countries, so that our analyses focussed on epidemic spread within the UK rather than including case importation. A few sporadic cases were identified through sentinel primary care testing rather than CC but could not be identified as such; however, there were only three such cases so the numbers were too small to affect our results. Sporadic cases were extracted from 16 February (the earliest date of onset for a sporadic case) to 6 March; cases peaked at six per day on 6 March and then declined, indicating that the FF100 was increasingly incomplete.

(ii) PHE and National Health Service (NHS) England compiled CC cases in the COVID-19 Hospitalisations in England Surveillance System (CHESS) database which were collected on 15–20 March (the seven cases reported on 13 and 14 March were ignored as the system was not fully operational then) [4].

We estimated the age-dependent risk of an infected case being admitted to CC by fitting our models to analyses of case data from China [5] and the United States (US) [6], with recently released Italian data [7] used in a sensitivity analysis (details in Supplement Chapter 1).

## Modelling

The same model was fitted independently to the incidence of patients admitted to CC based on (i) their date of symptom onset (for FF100 patients) or (ii) their date of admission (for CHESS patients, as date of symptom onset was not reported), using a Poisson likelihood with rate *Ae^Bt^*, where *A* is the initial number of cases on 16 February, *B* is the growth rate of the epidemic, *t* is time in days after 16 February and *e* is the exponential constant (see Supplement Chapter 4). Delays between symptom onset and reporting were accounted for by fitting a gamma distribution to time between symptom onset and CC admission for FF100 patients (see Supplement Chapter 2). When fitting modelled CC admissions to FF100, incidence was inflated by 18.6% compared with CC admissions fitted to CHESS, to adjust for the ratio of the population of the UK to that of England (the ratio of reported COVID-19 cases in the UK to that reported in England on 15–23 March would give a similar inflation factor of 19.8%).

*A* and *B* were sampled from their posterior distributions by using importance sampling; 10,000 parameter sets for both were drawn from uniform distributions between 0 and 1, and then resampled with replacement at a probability for each sample weighted by the likelihood of that parameter set. Projected CC cases were then divided by age-dependent risks of CC admission to estimate actual infections in the population (Supplement Chapter 1).

## Results

We estimated that each COVID-19 case admitted to CC reported in FF100 and CHESS corresponded to a median of 124 (95% credible interval (CrI): 81–11,500) and 120 (95% CrI: 76–46,600) infected individuals in the population, respectively, based on Chinese and US severity data [5,6]. 

The Figure shows the number of incident cases estimated on each day between 16 February and 23 March. On 23 March, 114,000 (95% CrI: 78,000–173,000) new cases and 258 (95% CrI: 220–319) CC reports are estimated to have occurred, with 527,000 (95% CrI: 362,000–797,000) cumulative cases since 16 February. The best fitting exponential growth rates were consistent with an epidemic doubling time of 2.8 days (95% CrI: 2.6–3.0). Assuming an exponentially distributed serial interval of 4 days [8] gave an (approximate) reproduction number of 2.0 (95% CrI: 1.9–2.1). If we assume a longer serial interval of 6 days that may be expected at the start of an epidemic, the reproduction number could be 2.5 (95% CrI: 2.4–2.6).

**Figure:**
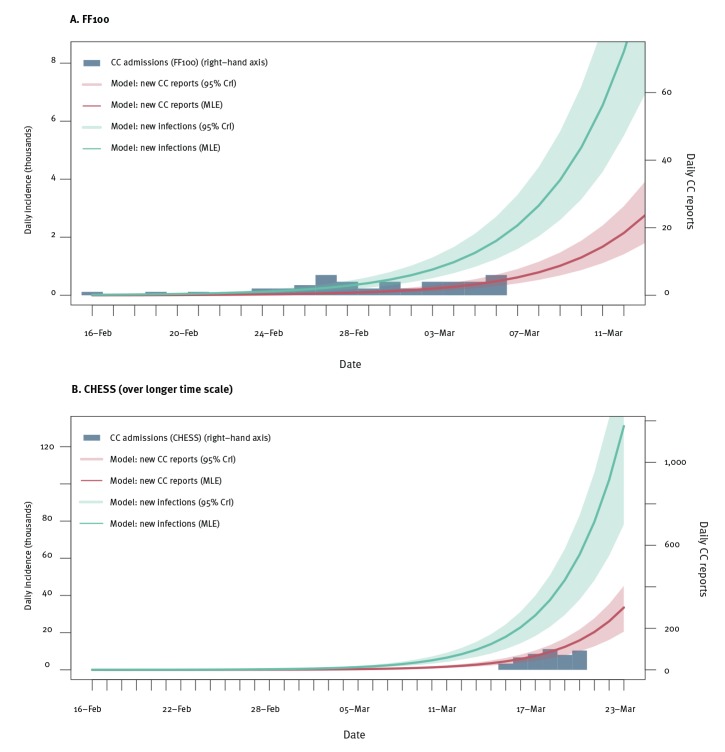
Model estimates of daily new COVID-19 infections and critical care reports in the initial phase of the epidemic compared with source data, United Kingdom, February–March 2020

CC: critical care; CHESS: COVID-19 hospitalisations in England surveillance system; FF100: first few hundred; MLE: maximum likelihood estimate; 95% CrI: 95% credible interval.

The curves corresponding to the MLE are in darker colours.

We performed sensitivity analyses on the datasets used for fitting, sensitivity of case detection and reporting, period of validity of the FF100 data and severity of COVID-19. Across the scenarios, the number of new infections on 23 March ranged from 95,000 (95% CrI: 62,000–182,000) to 143,000 (95% CrI: 95,000–228,000) (Supplement Chapter 3).

For validation, we compared the median total number of CC admissions from 16 to 23 March (with that period of time chosen to correspond to an average duration of CC stay of 8 days in Wuhan data [9]) with the prevalence of COVID-19 patients in CC on 23 March (i.e. number of patients reported to be in CC) according to NHS England’s situation report. Our model projected that 1,200 (95% CrI: 1,060–1,380) patients were in CC on 23 March, ca 68% more than the figure of 714 from the situation report when inflated to account for the larger UK population.

## Discussion

Our results suggest that hundreds of thousands of COVID-19 infections had occurred in the UK by the time the national lockdown of 23 March was implemented, with incidence doubling every 2.8 (95% CrI: 2.6–3.0) days. This suggests that only around 1% of infections were being detected and reported, since the official case count on 23 March was 6,650 [1]. This provides evidence that strict physical distancing was necessary to prevent health services from being overwhelmed.

However, these figures are still orders of magnitude below the scenarios from Lourenço et al. [10] that suggest that the majority of the UK population (66 million) could have already been infected by that date. The Lourenço scenarios producing these conclusions rely on severity pyramids that are much milder than those observed from Chinese [5], US [6] and Italian [7] data. Our own analyses indicate that severity is indeed a key driver of uncertainty in case estimates but across all scenarios, the majority of the UK population remained uninfected, and hence timely interventions to reduce physical contact could have a large impact. Importantly, our analyses relied on data from these countries only to establish the proportion of infected people who required critical care (which we expect to vary less between countries with reasonable healthcare access), and not for parameters likely to differ widely between countries such as the proportion of cases which are reported.

Our investigations revealed weaknesses in two early sources of CC surveillance (FF100 and CHESS). In particular, the number of cases reported in the two databases differ markedly, with the FF100 reporting far fewer CC cases (e.g. six cases on 6 March from FF100 compared with 100 on 18 March from CHESS). Our model projections suggest that the FF100 numbers are lower even after accounting for different periods of coverage and delays to reporting. Further analysis also suggested delays between the date of being reported as a case and the date on which a case is actually recorded in the FF100 database which made correcting for right-truncation of cases difficult (see Supplement Chapter 2). Also, the proportion of cases estimated to require CC treatment was substantially lower than observed in other countries (and hence the multiplication factors to convert CC cases to infections are higher) because of the young age profile of CC cases in both FF100 and CHESS. For example, 34% of CC patients were younger than 45 years in CHESS compared with only 12% in published US data up to 16 March [6]. This may reflect biases in CHESS reporting, differential propensity to test by age or a higher triage threshold for CC admission of older patients than in the US. 

The overall conclusion that COVID-19 daily incidence in the UK on 23 March was in the hundreds of thousands of infections and cumulative incidence around half a million appears to be robust even when challenged by a range of sensitivity analyses to account for different assumptions. Our model was fitted to data from early on in the epidemic (16 February) to just before the national lockdown (20 March). Furthermore, the order of magnitude is the same as data from situation reports that have less granularity but possibly better coverage. Lower numbers in the situation reports may in part reflect decreasing propensity to admit patients as CC beds become filled, in line with triage guidelines issued during the pandemic [9]. In addition, our model may have slightly overestimated cases if their rate of increase had slowed down even before the lockdown started. This highlights the need to triangulate case estimates from multiple surveillance streams whenever possible. Our investigations highlight the usefulness of CC surveillance in understanding epidemic dynamics and informing response measures, and hence the need for timely and complete reporting over the course of the epidemic.

## LSHTM Centre for Mathematical Modelling of Infectious Disease COVID-19 working group

Arminder K Deol, Hamish Gibbs, Kathleen O'Reilly, Graham Medley, Joel Hellewell, Alicia Rosello, Billy J Quilty, Charlie Diamond, Petra Klepac, Amy Gimma, Rosalind M Eggo, Megan Auzenbergs, Samuel Clifford, Gwen Knight, Sebastian Funk, Anna Foss, Fiona Sun, Jon C Emery, Kiesha Prem, Yang Liu, Kevin van Zandvoort, Christopher I Jarvis, James D Munday, Adam J Kucharski, Carl A B Pearson, Timothy W Russell, Nikos I Bosse, Stefan Flasche, Rein M G J Houben, Simon R Procter, Nicholas G. Davies.

## Supplementary Data

312000 Supplement
