# Supplementary material for: Estimating number of cases and spread of coronavirus disease (COVID-19) using critical care admissions, United Kingdom, February to March 2020
Source: Euro Surveill. 2020 May 7;25(18):2000632. doi: 10.2807/1560-7917.ES.2020.25.18.2000632 (PMC7219029; doi:10.2807/1560-7917.ES.2020.25.18.2000632)
Supplement: Supplement [file 20-00632_supplement-JIT.pdf]

Supplemental material to "Estimating number of cases and spread of Coronavirus disease 2019 (COVID-19) in the United Kingdom using critical care admissions, February to March 2020"

This supplementary material is hosted by Eurosurveillance as supporting information alongside the article "Estimating number of cases and spread of coronavirus disease (COVID-19) using critical care admissions, United Kingdom, February to March 2020", on behalf of the authors, who remain responsible for the accuracy and appropriateness of the content. The same standards for ethics, copyright, attributions and permissions as for the article apply. Supplements are not edited by Eurosurveillance and the journal is not responsible for the maintenance of any links or email addresses provided therein.

1. Estimating the age-dependent proportion of infected people who are admitted to critical care

To estimate the proportion of COVID-19 infected people who are admitted to CC, three data sources were used: (a) estimates by Verity et al. [1] from case data in China and estimates of infection prevalence from exported cases, that were analysed to estimate the age-dependent proportion of infected cases that were hospitalised, (b) estimates by the Centres for Disease Control and Prevention (CDC) (Bialek et al. [2]) from case data in the USA on the age-dependent proportion of reported cases that were hospitalised or admitted to ICU, and (c) estimates from Riccardo et al. [3] from case data in Italy on the age-dependent proportion of reported cases that were hospitalised or admitted to ICU.

The CDC analysis contained two scenarios: a low scenario with all reported cases as the denominator, and a high scenario where only those with known hospitalisation status were used in the denominator. The CDC low scenario was used for our main analysis, but sensitivity analyses were conducted using the CDC high scenario and the Italian data.

Since these data were only available by age group, linear (r = M + Na), exponential (r = Me^{Na}) or logistic ($r = M/(1+\exp(-N(a-P))$) models (where r is the risk of hospitalisation/ICU admission, a is age in years and M, N and P are inferred parameters) were fitted to these

data in order to determine risks by single year of age. To infer *M*, *N* and *P*, Bayesian updating with a Binomial likelihood function was used, taking into account the actual number of cases and denominators corresponding to each age group. *M*, *N* and *P* were sampled from their posterior distributions by using importance sampling; 10,000 parameter sets for both were drawn from uniform distributions and then resampled with replacement at a probability for each sample weighted by the likelihood of that parameter set. To determine the range of the uniform distribution, sampling was first conducted over very broad priors, constructed from the distributions in Table S1 below. After resampling, the ranges of parameters in the resampled sets were then determined – these ranges were more likely to contain parameter sets with a high likelihood of fitting data. We hence sampled a second time from uniform distributions over these ranges to obtain our final posterior distributions.

Function	M	N	P
Linear	U[0,0.1]	U[0,0.01]	
Exponential	U[0,0.1]	U[0,0.1]	
Logistic	U[0,1]	U[0,1]	U[0,100]

Table S1. Uniform distributions from which the fitted parameters were sampled; U[*a*,*b*] denotes a uniform distribution with minimum *a* and maximum *b*.

Linear, exponential and logistic models were fitted to the data, and the model was selected for which the maximum likelihood estimate had the lowest Akaike Information Criterion, $AIC = -2 \log_e(MLE) + 2k$, where *MLE* is the maximum of the likelihood function and k is the number of parameters. A logistic model was chosen for most data sets, apart from a linear model for the *reported:hospitalised* (CDC high scenario) and exponential model for the *reported:hospitalised* (CDC low scenario).

These data were then combined to estimate the risk of ICU admission in infected patients using the following formula:

P(admitted to ICU | infected) = P(hospitalised | infected) x P(admitted to ICU | reported case) / P(hospitalised | reported case)

We assumed that this was the same as the risk of CC admission. These "multipliers" were then used to calculate the estimated number of infected people in the UK given an estimate of the number of COVID-19 CC admissions according to the equations below:

Let $A_{FF100}(a)$ and $A_{CHESS}(a)$ be the number of CC cases aged *a* years in the FF100 and CHESS datasets respectively, and P(*a*) be the probability of a person infected with COVID-19 at age *a* being admitted to CC based on our analysis of Chinese, US and Italian dataset. Then m(*a*) = $P(a)^{-1}$ is the corresponding multiplier giving the number of infected people of age *a* when the number of CC admissions of age a is P(*a*).

Let m_{FF100} and m_{CHESS} the age-adjusted multiplier to estimate the number of infected people given the number of CC admissions for the FF100 and CHESS datasets respectively. Then $m_{FF100} = \frac{\sum_{a=0}^{99} A_{FF100}(a)m(a)}{\sum_{a=0}^{99} A_{FF100}(a)}$ and $m_{CHESS} = \frac{\sum_{a=0}^{99} A_{CHESS}(a)m(a)}{\sum_{a=0}^{99} A_{CHESS}(a)}$.

Then:
Number of infected people on a given day using FF100 data = Number of CC admissions with symptom onset on that day x m_{FF100}
Number of infected people on a given day using CHESS data = Number of CC admissions with symptom onset on that day x m_{CHESS}

Children below 15 years were removed from UK datasets when calculating multipliers since there were no ICU admissions in these age groups in the other datasets – we believe that the over-representation of younger people in the CHESS dataset in particular reflects biases in reporting (see Discussion of main paper).

Figures S1.1 – S.14 below show the posterior distributions for the best fitting models at each stage of the process.

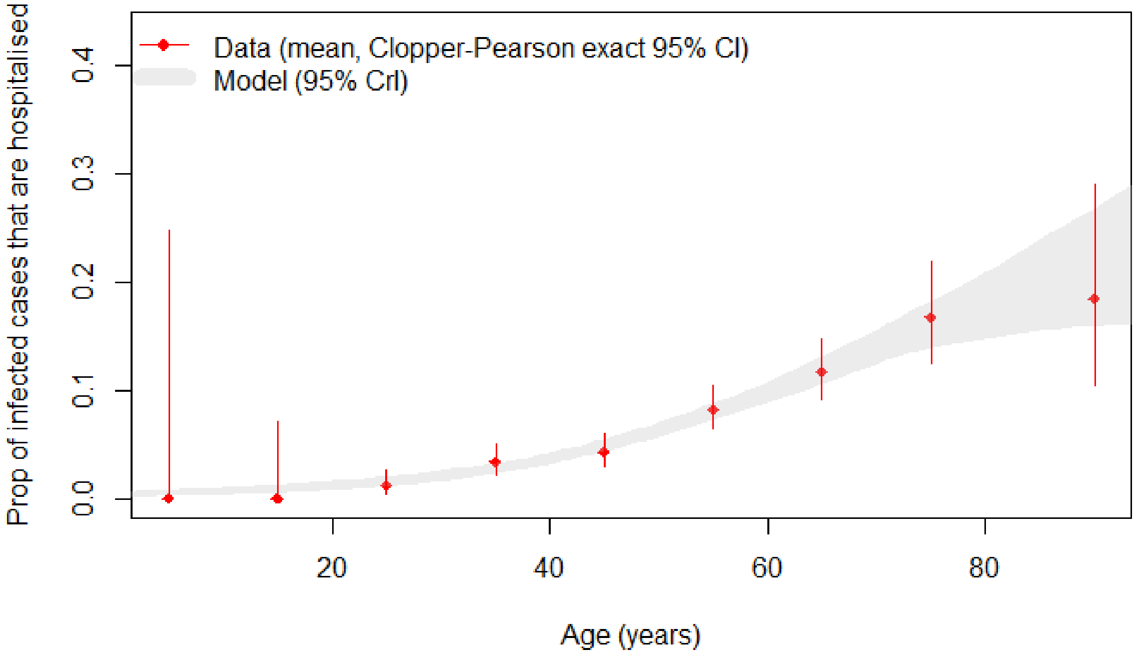

Figure S1.1. Age-dependent proportion of infected cases that are hospitalised, showing data from Verity et al. [1] and the 95% credible interval of an exponential model using the posterior distribution of parameter sets. (AIC: linear 77.0, exponential 50.6, logistic 44.6)

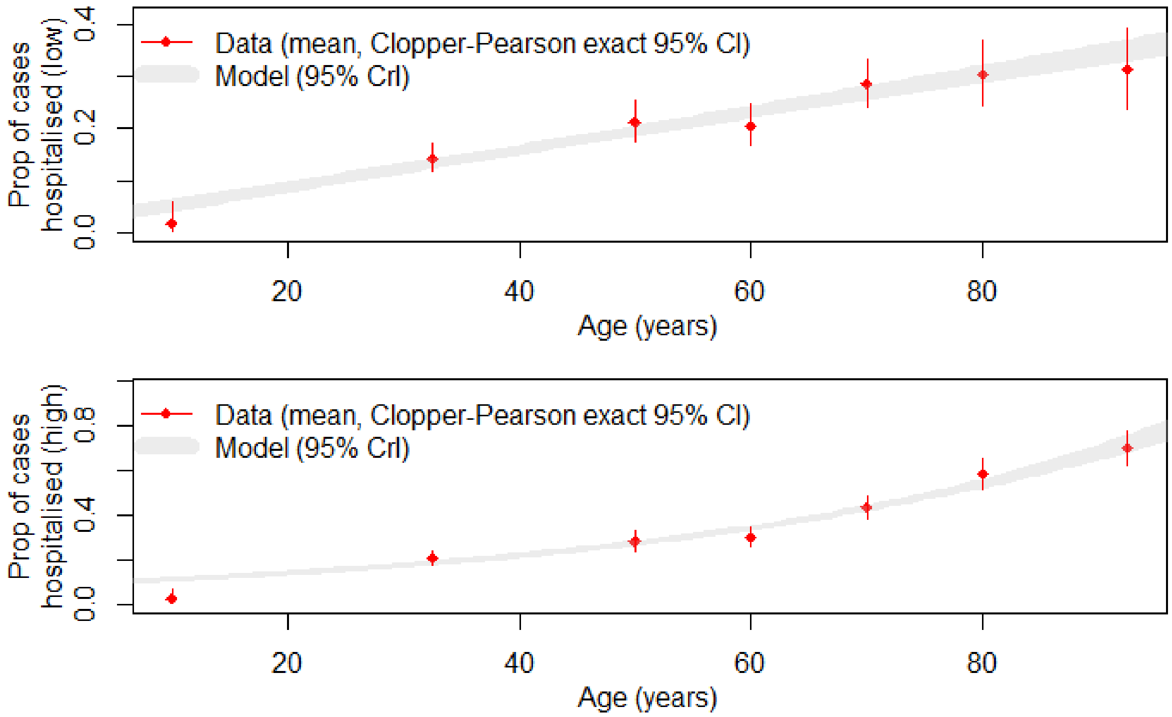

Figure S1.2. Age-dependent proportion of reported cases that are hospitalised, showing data from Bialek et al. [2] for the low (top) and high (bottom) scenarios, and the 95% credible interval of a linear model using the posterior distribution of parameter sets. (AIC: linear 50.8, exponential 62.2, logistic 54.0 for low scenario; linear 75.6, exponential 65.2, logistic 67.3 for high scenario)

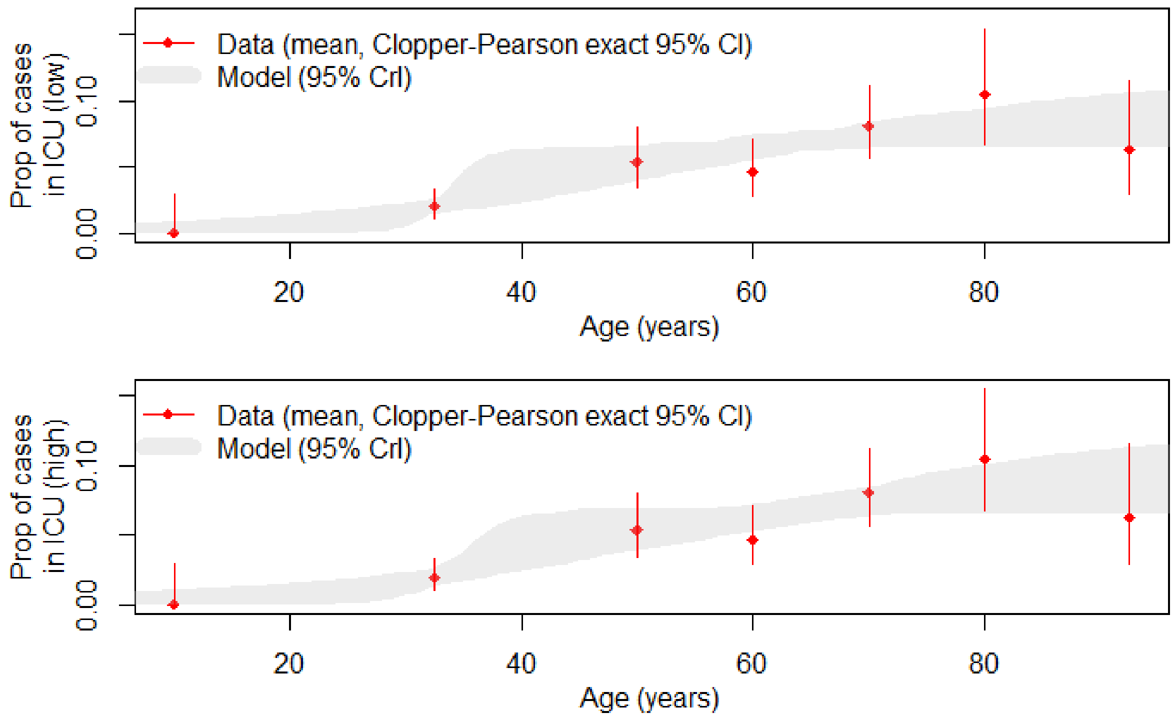

Figure S1.3. Age-dependent proportion of reported cases that are admitted to ICU, showing data from Bialek et al. [2] for the low (top) and high (bottom) scenarios, and the 95% credible interval of a logistic model using the posterior distribution of parameter sets. (AIC: linear 43.8, exponential 46.1, logistic 40.6 for low scenario; linear 43.9, exponential 46.1, logistic 40.8 for high scenario)

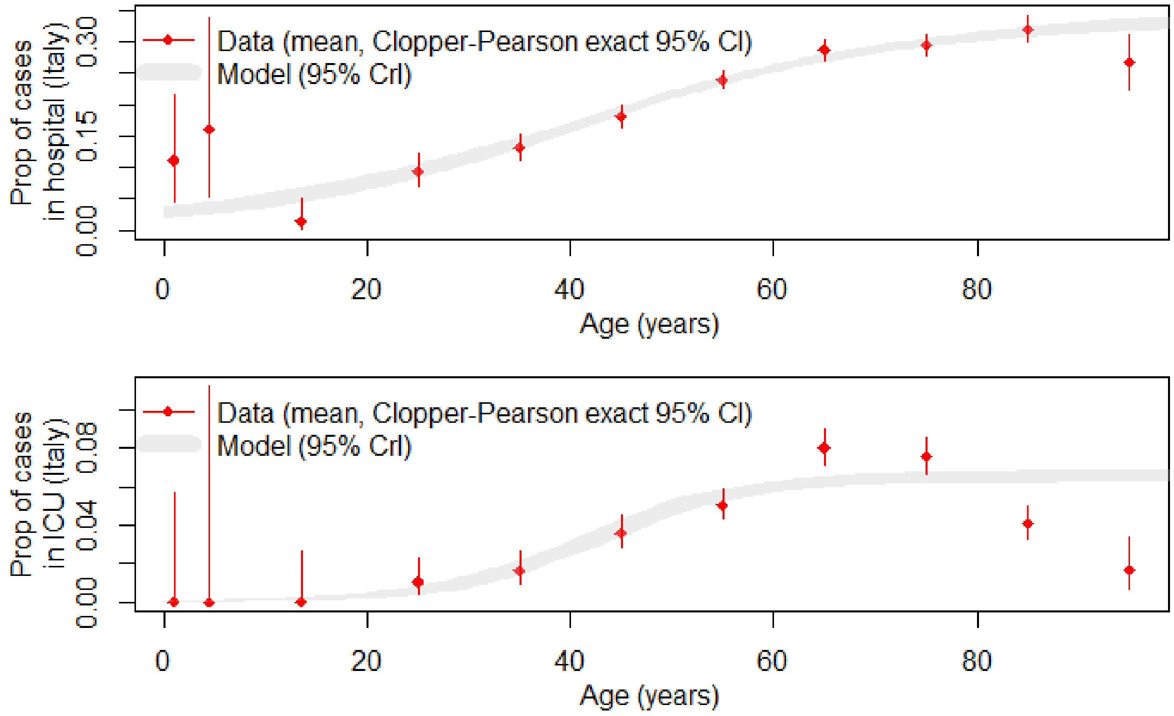

Figure S1.4. Age-dependent proportion of reported cases that are admitted to hospital (top) and to ICU (bottom), showing data from Riccardo et al. [3] and the 95% credible interval of a logistic model using the posterior distribution of parameter sets. (AIC: linear 43.8, exponential 46.1, logistic 40.6 for low scenario; linear 43.9, exponential 46.1, logistic 40.8 for high scenario)

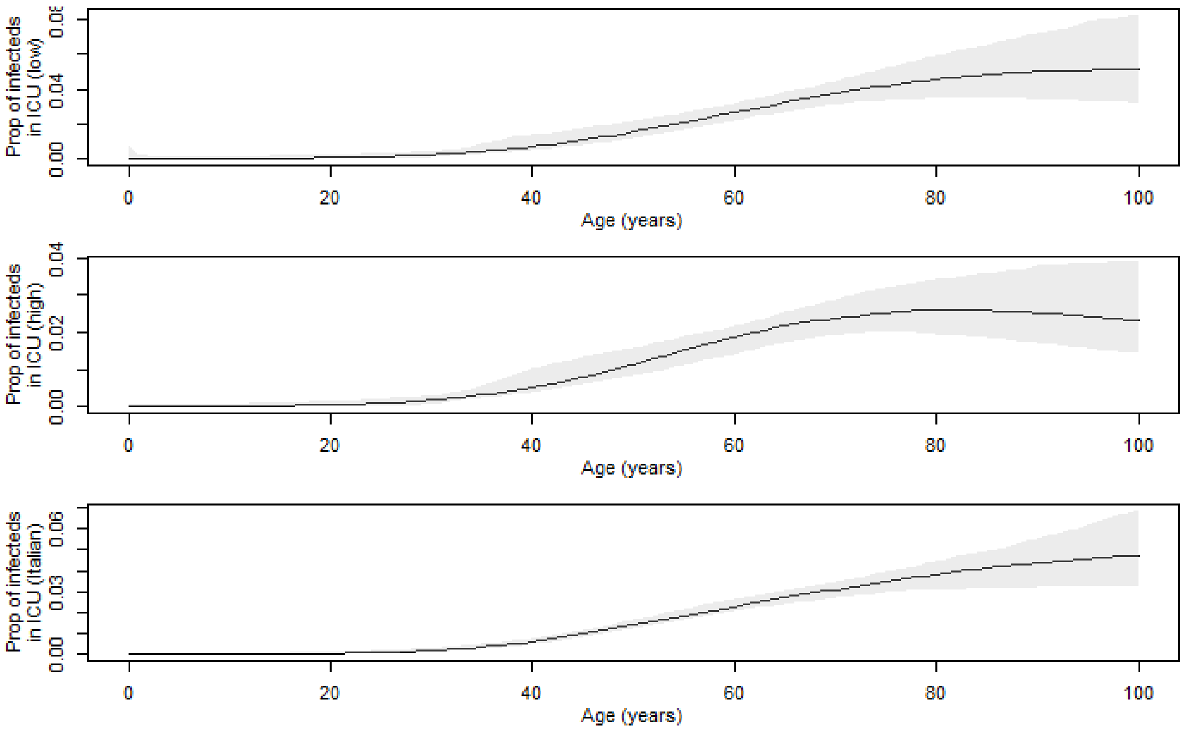

Figure S1.4. Age-dependent proportion of infected patients who are admitted to ICU, based on best fitting scenarios from the combination of models fitted to CDC low (top), CDC high (middle) and Italian (bottom) scenarios. The line with the darker colour is the best fitting (maximum likelihood) scenario.

2. Estimating delays between onset of symptoms and reporting

Extraction of data

To estimate the delay between onset of symptoms and being reported as a CC admission, we extracted the observed time difference for 70 cases in the FF100 dataset (extracted on 31 March 2020) who were admitted to CC (i.e. all cases in the FF100 that were labelled as sporadic, and have both a symptom onset and a reporting date that was later than the date of symptom onset). The reporting date was assumed to be the date they were admitted to CC, since in all cases it was later than the date of hospital admission.

The distribution of time differences is shown in Figure S2 below, this has a median of 6.5 days (interquartile range 4.2 - 10, 95% interval 1 - 20).

Fit to data

The data were fitted using a discretised Gamma function (with probability distribution function $\gamma(d) = \Gamma(d+1) - \Gamma(d)$, where d is the delay in days and $\Gamma(d)$ is the cumulative distribution function of a continuous Gamma function). To infer the shape and size parameters of $\gamma(d)$, Bayesian updating was used with both parameters drawn from uniform priors on the range [0,10]. The posterior distribution of the parameters was sampled from using importance sampling; parameter sets were sampled from their priors with replacement, with probability of sampling each parameter set weighted by the likelihood of the set. The posterior Gamma distribution is shown in Figure S2 below.

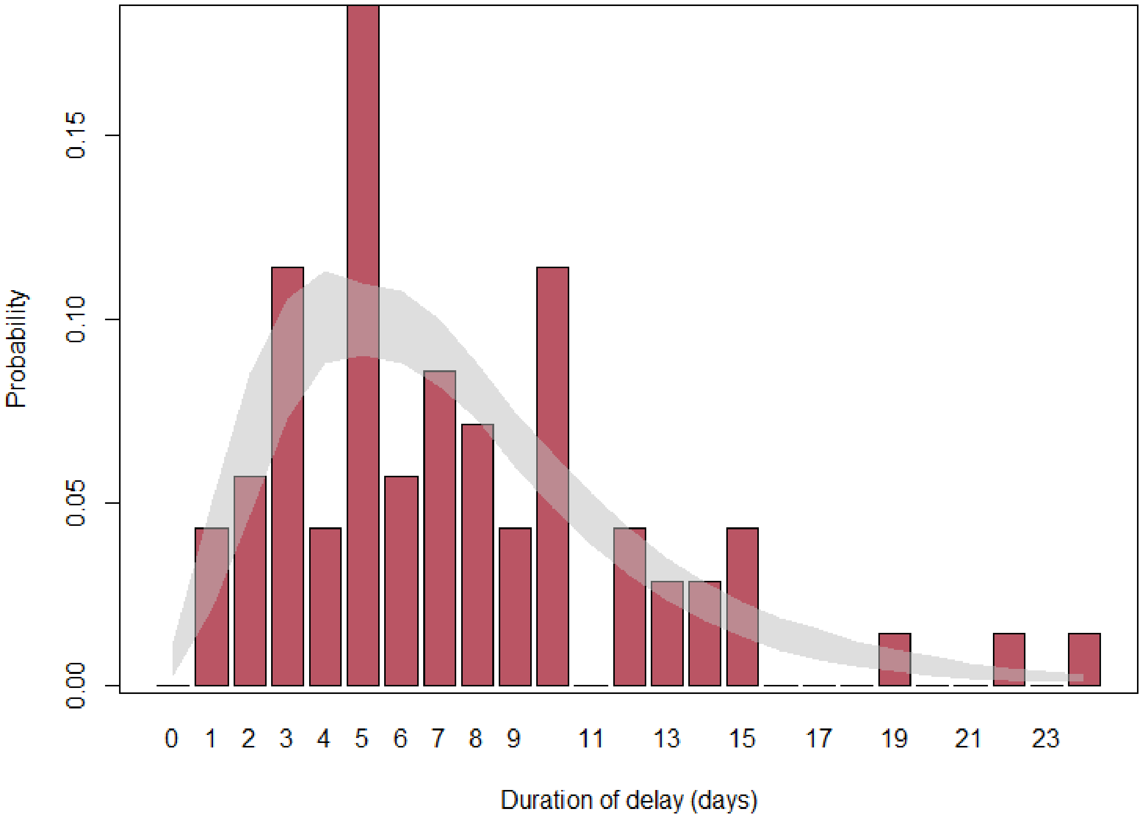

Figure S2. Observed delay between onset of symptoms and being reported as a CC admission for patients in FF100 with both dates, and 95% credible interval for a gamma distribution using the posterior distribution of parameter sets.

Use in the model

The delay distribution was applied to the distribution of infections over time to generate a distribution of reported CC cases over time.

Issues around truncation bias

Note that the observed distribution of delays is subject to right truncation, i.e. in the middle of an epidemic, some patients will not yet be recorded as CC admissions, due to the delay between onset of disease and reporting. This reporting delay means that, on any particular day, the data available on onset cases will exclude those cases which have onset but have not yet been reported. We therefore see only a portion of the true epidemic curve. It is possible to adjust for this bias by nowcasting - using the delay we have observed for already reported cases to estimate how many additional, onset-not-yet-reported cases are ongoing at any point in time. Particularly early on in the epidemic when incidence is increasing exponentially, the observed delay distribution will be biased downwards since only patients with shorter delays will already have been reported to the system. As time goes on and we observe enough of the epidemic to capture the longest reasonable delay, this bias will diminish and the delays can be estimated directly but will likely have changed from the early delays.

However, with these data sources such a correction is difficult to do, not only because of the small sample size, but also because there are in fact two sources of right-truncation: (i) the delay from onset of symptoms to being reported as a CC case as just described (a “patient” delay) and (ii) the delay from being reported as a CC case to actually being available in the dataset to national researchers (a “system” delay, for example varying by the reporting trust). Decreasing effort in maintaining the dataset also likely contributed to the tailing-off of reported cases in the FF100; whether due to delay or poor completion, some cases reported on those days will never be added to this dataset. This would bias counts of CC admissions which is one reason why the only counts up to 6 March in the FF100 were used for model fitting; however for the delay distribution we used all patients with a date of reporting for CC admission up to the last complete dataset (31 March).

3. Sensitivity analyses

We performed sensitivity analyses by considering the following alternative scenarios:

- Dataset used. Restricting the analysis to the CHESS dataset only (i.e. not using the FF100 dataset).
- Altering the sensitivity of COVID-19 detection in CC to 75%; this could reflect both testing sensitivity (e.g. poor sample collection) or incomplete reporting of test positives.
- Changing the period of validity of the FF100 database to the full period instead of just to 12 March.
- Using the CDC "high" scenario for severity of COVID-19
- Using the Italian dataset instead of the CDC dataset.
- Including individuals under 15 years old in the CHESS data set when calculating the proportion of infected cases who need CC.

The Table below shows the number of new infections on 23 March, cumulative infections from 16 February - 23 March and epidemic doubling time for each scenario. Figures show median and 95% credible intervals.

Scenario	New infections on 23 March (000s)	Cumulative infections by 23 March (000s)	Exponential growth rate (days)	Epidemic doubling time (days)
**Base case**	114 (78, 173)	527 (362, 797)	0.25 (0.23, 0.27)	2.82 (3.04, 2.57)
**Using CHESS only**	95 (62, 182)	470 (316, 875)	0.22 (0.22, 0.25)	3.12 (3.22, 2.83)
**Low detection sensitivity**	129 (87, 213)	601 (408, 962)	0.24 (0.22, 0.26)	2.88 (3.10, 2.71)
**Full FF100 period**	96 (63, 150)	455 (288, 687)	0.24 (0.22, 0.26)	2.90 (3.16, 2.68)
**CDC "high" multipliers**	142 (95, 235)	669 (459, 999)	0.24 (0.22, 0.26)	2.87 (3.15, 2.63)

**Italian multipliers**	143 (95, 228)	663 (457, 988)	0.24 (0.22, 0.26)	2.85 (3.08, 2.64)
**Including <15 year olds**	116 (82, 209)	514 (362, 878)	0.25 (0.25, 0.27)	2.72 (2.77, 2.56)

4. Model specification

Variables

a_d = number of critical care COVID-19 admissions with symptom onset on day *d* after 16 February 2020

b_d = number of critical care COVID-19 admissions on day *d* after 16 February 2020

γ_d = distribution of delay between symptom onset and being reported as a critical care case

F_d = number of COVID-19 infections in FF100 that result in critical care admission with symptom onset on day *d* after 16 February 2020

C_d = number of critical care admissions in CHESS admitted on day *d* after 16 February 2020

ρ_1 = proportion of infected cases that become critical care admissions, age-adjusted using FF100 data

ρ_1 = proportion of infected cases that become critical care admissions, age-adjusted using CHESS data

Equations

$$a_d = Ae^{Bd}$$

$$b_d = \sum_{t=0}^{d} a_t \gamma_{d-t}$$

$$Likelihood(A, B|F, C) = \prod_{t=0}^{D} Pois(x = F_d; \lambda = a_d) \prod_{t=0}^{D} Pois\left(x = C_d; \lambda = b_d \frac{\rho_1}{\rho_2}\right)$$

Where A and B are parameters to be fitted, Likelihood(A,B|F,C) is the likelihood function for A and B given the observed values of F_d and C_d, and *Pois*(X = *x*; λ) = $\lambda^x e^{-\lambda} / x!$ is the Poisson probability mass function of X.

References

1. Verity R, Okell LC, Dorigatti I, Winskill P, Whittaker C, Imai N, et al. Estimates of the severity of coronavirus disease 2019: a model-based analysis. Lancet Infect Dis. 2020;0. doi:10.1016/S1473-3099(20)30243-7.

2. Bialek S, Boundy E, Bowen V, Chow N, Cohn A, Dowling N, et al. Severe Outcomes Among Patients with Coronavirus Disease 2019 (COVID-19) — United States, February 12–March

16, 2020. MMWR Morb Mortal Wkly Rep. 2020;69:343–6. doi:10.15585/mmwr.mm6912e2.
3. Riccardo F, Ajelli M, Andrianou X, Bella A, Manso M Del, Fabiani M, et al. Epidemiological characteristics of COVID-19 cases in Italy and estimates of the reproductive numbers one month into the epidemic. medRxiv. 2020;:2020.04.08.20056861. doi:10.1101/2020.04.08.20056861.
